# Metabolic correlates of subcutaneous and visceral abdominal fat measured by ultrasonography: a comparison with waist circumference

**DOI:** 10.1186/s12937-015-0120-2

**Published:** 2016-01-06

**Authors:** Simona Bertoli, Alessandro Leone, Laila Vignati, Angela Spadafranca, Giorgio Bedogni, Angelo Vanzulli, Elena Rodeschini, Alberto Battezzati

**Affiliations:** 1 Department of Food, Environmental and Nutritional Sciences (DEFENS), International Center for the Assessment of Nutritional Status (ICANS), University of Milan, Via Botticelli 21, 20133 Milano, Italy; 2 Division of Radiology, Ospedale Niguarda Cà Granda, Milan, Italy

**Keywords:** Epidemiology, Cross-sectional study, Visceral fat, Subcutaneous fat, Waist circumference, Ultrasonography, Metabolic syndrome, Liver enzymes, Uric acid

## Abstract

**Background:**

The relative contribution of visceral (VAT) and subcutaneous (SAT) adipose tissue to cardiometabolic disease is controversial. The aim of this study was to evaluate whether dissecting abdominal fat in VAT and SAT using US may detect stronger and more specific association with MS, MS components, hyperuricemia and altered liver enzymes compared to waist circumference.

**Methods:**

We performed a cross-sectional study on 2414 subjects aged 18 to 66 years (71 % women) followed at the International Center for the Assessment of Nutritional Status (ICANS, Milan, Italy). VAT and SAT were measured using ultrasonography. Multivariable logistic regression controlling for age and gender was used to evaluate the association of the parameters of interest (waist circumference (WC), VAT, SAT and VAT + SAT) with the MS (international harmonized definition), its components (high triglycerides, low HDL, high blood pressure, high glucose), high uric acid (≥7 mg/dl), high alanine transaminase (ALT, ≥ 30 U/l) and high gamma-glutamyl-transferase (GGT, ≥ 30 U/l).

**Results:**

VAT was independently associated with all the outcomes of interest, while SAT was independently associated with MS and only with high blood pressure and high ALT when we considered the single parameters of MS and NAFLD. VAT had the strongest association with high triglycerides, high ALT and high GGT. The VAT + SAT association had the strongest association with MS. WC had the strongest association with low HDL and high blood pressure. VAT and WC were similarly associated to high glucose and high uric acid.

**Conclusion:**

US-determined VAT and SAT are both independently associated with MS. Moreover, to our knowledge, we are the first to show that VAT, being associated to all of the MS components in addition to hyperuricemia and altered liver enzymes, performs equally or better than WC except for high blood pressure and low HDL.

**Electronic supplementary material:**

The online version of this article (doi:10.1186/s12937-015-0120-2) contains supplementary material, which is available to authorized users.

## Background

Abdominal fat distribution has been recognized as an important risk factor for cardiometabolic disease (CMD) [1]. Increased levels of abdominal visceral adipose tissue (VAT) are associated with the metabolic syndrome (MS), type 2 diabetes mellitus (T2DM), cardiovascular disease [2–6], non-alcoholic fatty liver disease (NAFLD) [7, 8] and hyperuricemia [9]. On the other hand, the contribution of subcutaneous adipose tissue (SAT) to CMD is still debated [10].

Waist circumference (WC) is often used as surrogate measure of abdominal fat, but it cannot separate the effect of VAT from that of SAT [11]. Computed tomography (CT) and magnetic resonance imaging (MRI) are the reference methods for the assessment of VAT and SAT [12, 13] and CT-measured VAT is associated with MS more strongly than WC [2]. However, because they are expensive and because CT exposes to ionizing radiation, MRI and CT cannot be used in large epidemiological studies. Ultrasonography (US) offers a cheap and non-invasive alternative to MRI and CT [14–18]. US has been used to measure abdominal VAT and SAT since the early 90s and offers accurate and reproducible estimates provided that standardized measurement protocols are used [12–19].

US-determined VAT is associated with insulin resistance, several CMD and NAFLD risk factors (e.g., fasting glucose, lipid profile, blood pressure and liver enzymes) [20–27]. However, the contribution of SAT to these outcomes is controversial [20, 25–27]. It is also not clear whether US-measured VAT and SAT are associated more strongly with MS and its components, altered liver enzymes and hyperuciremia than is WC.

Therefore, the aim of the present cross-sectional study, performed in a large sample of outpatients followed at a Nutritional Research Center was to evaluate whether dissecting abdominal fat in VAT and SAT using US may detect stronger and more specific association with MS, MS components, hyperuricemia and altered liver enzymes compared to waist circumference.

## Methods

### Subjects

2414 Caucasian subjects (1714 women, 71 %) were consecutively studied at the International Center for the Assessment of Nutritional Status (ICANS, Milan, Italy) between September 2010 and June 2012. All subjects were enrolled because of their interest to undergo a structured nutritional assessment. Inclusion criteria were: 1) age ≥ 18 years; 2) body mass index (BMI) ≥ 18.5 kg/m^2^. Exclusion criteria were: 1) acute disease, e.g., influenza; 2) heart, pulmonary, gastrointestinal, neurological or neoplastic disease; 3) use of medications known to cause lipodystrophy, e.g., steroids and antiretroviral agents); 4) presence of scars in the measurement area of VAT and SAT. On the same morning, the subjects underwent a medical interview, an anthropometric assessment, a measurement of blood pressure, an abdominal US, and blood sampling. The study was performed in accordance with the Declaration of Helsinki and the subjects gave their written informed consent. The local Ethical Committee approved the study procedures.

### Clinical and anthropometric assessment

A detailed medical interview was performed and the use of any drug was recorded. Weight and height were measured following international guidelines [28]. BMI was calculated as weight (kg) / height (m)^2^ and obesity was classified following the WHO guidelines [29]. WC was measured at the midpoint between the last rib and the iliac crest [29]. Systolic and diastolic blood pressure were measured following the JNC-7 guidelines [30].

### Abdominal ultrasonography

Abdominal US was performed on fasting subjects by the same operator using a Logiq 3 Pro instrument equipped with a 3.5 MHz convex-array probe and with a 7.5 MHz linear probe (GE Healthcare, Milwaukee, WI, USA). VAT and SAT were measured 1 cm above the umbilicus. The examination was performed at end-expiration and applying the same probe pressure for all subjects. SAT was measured with the 7.5 MHz linear probe as the distance between the epidermis and the external face of the *rectus abdominis* muscle; VAT was measured with the 3.5 MHz convex-array probe as the distance between the anterior wall of the aorta and the posterior surface of the *rectus abdominis* muscle [14]. Each measurement was performed 3 times and the mean of the 3 measurements was used for analysis. The within-day intra-operator coefficient of variation (CV) for repeated measures of VAT and SAT in our laboratory is 0.8 %.

### Laboratory assessment

Fasting blood samples were drawn between 8:30 and 9:00 AM and analyzed in the same morning at the ICANS laboratory. Glucose, triglycerides, HDL-cholesterol, alanine transaminase (ALT), gamma-glutamyl-transferase (GGT) and uric acid were measured by means of an enzymatic method (Cobas Integra 400 Plus, Roche Diagnostics, Rotkreuz, Switzerland), with intra-and inter-assay CVs < 2 %. High ALT was defined as ALT ≥ 30 U/L and high GGT as GGT ≥ 35 U/L [31]. High uric acid was defined as uric acid ≥ 7 mg/dl, i.e., the upper normal limit of the ICANS laboratory.

### Metabolic syndrome

MS was diagnosed using the harmonized international definition [32]. In detail, high WC was defined as WC ≥ 102 cm in men and ≥ 88 cm in women; low HDL as HDL < 40 mg/dl in men and < 50 mg/dl in women; high triglycerides as triglycerides ≥ 150 mg/dl; high blood pressure as systolic blood pressure ≥ 130 mmHg or diastolic blood pressure ≥ 85 mmHg or treatment with pressure-lowering drugs; and high glucose as glucose ≥ 100 mg/dl or treatment with glucose-lowering drugs. Type 2 diabetes mellitus (T2DM) mellitus was defined as blood glucose ≥ 126 mg/dl or treatment with glucose-lowering drugs.

### Statistical analysis

Most continuous variables were not normally distributed and all are reported as 50^th^, 25^th^ and 75^th^ percentiles. Categorical variables are reported as numbers and percentages. All continuous variables besides age were winsorized using a tail of 0.01. This implies that values under the 1^st^ or over the 99^th^ internal percentile were put equal to the 1^st^ or 99^th^ internal percentile, respectively. Winsorization limits the influence of outliers, a strategy that is important to increase the generalizability of regression models [33, 34]. The association of the 4 continuous variables (WC, VAT, SAT, all in cm) or combinations of variables (VAT and SAT, all in cm) with the 8 dichotomous outcomes (high triglycerides, low HDL, high blood pressure, high glucose, MS, high uric acid, high ALT and high GTT, 0 = no; 1 = yes) was evaluated using logistic regression models with age (continuous, years/10) and sex (discrete, 0 = female; 1 = male) as covariates [35]. We did not evaluate the association of WC and MS because WC is included in the definition of MS [32]. Multivariable fractional polynomials were used to model non-linear associations of continuous predictors with the outcomes [36]. Using this approach, we found that an inverse-transformation of WC (WC^−1^) and a log_e_-transformation of VAT (log_e_vat) ensured linear logits and better fits for all models. Such transformed values of WC and VAT were therefore used for the final analysis. We used the Hosmer-Lemeshow (HL) test and the standardized Pearson test to assess the goodness of fit (GOF) of the models [35]. In the few instances where GOF was rejected by the HL test, it was not rejected by the more powerful standardized Pearson test. In view of the available knowledge, we consider this to be a sufficient proof of the acceptable fit of all the models [35]. We used mcfadden pseudo-R^2^ and the area under the ROC curve (AUC-ROC) as measures of predictive ability [35]. To perform a relative comparison of the models we used the Bayesian information criterion (BIC) [37, 38]. “Weak evidence” in favor of the model with the lower BIC is said to exist when the BIC difference (ΔBIC) is ≤ 2; “positive evidence” when 6 > ΔBIC > 2; “strong evidence” when 6 ≤ ΔBIC < 10; and “very strong evidence” when ΔBIC > 10 [38, 39]. If ΔBIC < 10, and PSEUDO-R^2^ and AUC-ROC were not different, we defined the models similarly associated to the outcome considered.

## Results

Table 1 gives the measurements of the 2414 study subjects. They were aged 18 to 66 years and were mostly women (*n* = 1714, 71 %).

**Table 1:** Measurements of the study subjects

	Females (*n* = 1714)			Males (*n* = 700)			Total (*n* = 2414)		
	P_50_	P_25_	P_75_	P_50_	P_25_	P_75_	P_50_	P_25_	P_75_
Weight (kg)	72.6	64.2	83.3	92.3	83.5	103.6	78.1	67.4	91.2
Height (m)	1.62	1.57	1.66	1.75	1.71	1.80	1.65	1.59	1.72
BMI (kg/m^2^)	27.8	24.6	31.9	30.0	27.6	33.4	28.6	25.4	32.4
WC (cm)	90.5	82.0	100.0	105.5	98.0	114.2	95.0	85.1	105.5
VAT (cm)	4.2	3.0	5.8	7.1	5.4	9.0	4.9	3.4	7.1
SAT (cm)	2.8	2.1	3.7	2.7	2.0	3.7	2.8	2.0	3.7
Glucose (mg/dl)	91	85	98	97	91	105	93	87	100
Triglycerides (mg/dl)	82	61	116	121	85	171	91	66	131
Cholesterol (mg/dl)	211	183	239	212	187	240	211	184	240
HDL-cholesterol (mg/dl)	62	53	72	46	39	54	58	47	69
LDL-cholesterol (mg/dl)	128	105	153	138	115	162	130	107	155
ALT (U/l)	17	13	24	31	22	41	20	15	30
GGT (U/l)	16	12	23	31	21	46	19	13	30
Uric acid (mg/dl)	4.3	3.6	5.0	6.2	5.4	6.9	4.7	3.9	5.8
Systolic BP (mm Hg)	120	110	130	130	120	140	120	115	130
Diastolic BP (mm Hg)	75	70	80	80	80	90	80	70	85

*BMI* Body Mass Index, *WC* waist circumference, *VAT* visceral adipose tissue, *SAT* subcutaneous adipose tissue, *ALT* alanine transaminase, *GGT* gamma-glutamyl-transferase, *BP* blood pressure

Table 2 reports the frequency of MS, MS components (high waist circumference, high triglycerides, low HDL, high blood pressure and high glucose), high uric acid, high ALT and high GGT. Table 2 also reports the frequency of subjects treated with glucose-lowering, triglyceride-lowering, cholesterol-lowering and blood pressure-lowering drugs. Following current recommendations [32], subjects taking glucose-lowering drugs were classified as having high glucose and those taking blood pressure-lowering drugs as having high blood pressure. 59 % of the subjects had high WC, 19 % high triglycerides, 20 % low HDL, 50 % high blood pressure, 26 % high glucose, 29 % MS, 8 % high uric acid, 25 % high ALT, and 18 % high GGT. Less than 1 % of subjects was taking glucose-lowering or triglyceride-lowering drugs, 4 % cholesterol-lowering drugs, and 17 % blood-pressure lowering drugs. T2DM was diagnosed in 3 % of subjects.

**Table 2:** Age, nutritional status, components of metabolic syndrome, high uric acid, high ALT, high GGT in the study subjects according to sex

	Females		Males		Total	
	N	%	N	%	N	%
Age (years)						
18–29	204	11.9	67	9.6	271	11.2
30–39	371	21.6	153	21.9	524	21.7
40–49	538	31.4	215	30.7	753	31.2
50–59	392	22.9	153	21.9	545	22.6
60–69	209	12.2	112	16.0	321	13.3
Total	1714	100.0	700	100.0	2414	100.0
Weight status						
Normal	476	27.8	57	8.1	533	22.1
Overweight	652	38	286	40.9	938	38.9
Obesity class 1	356	20.8	239	34.1	595	24.6
Obesity class 2	151	8.8	85	12.1	236	9.8
Obesity class 3	79	4.6	33	4.7	112	4.6
Total	1714	100.0	700	100.0	2414	100.0
High waist circumference						
No	726	42.4	262	37.4	988	40.9
Yes	988	57.6	438	62.6	1426	59.1
Total	1714	100.0	700	100.0	2414	100.0
High triglycerides						
No	1497	87.3	466	66.6	1963	81.3
Yes	217	12.7	234	33.4	451	18.7
Total	1714	100.0	700	100.0	2414	100.0
Low HDL						
No	1405	82.0	525	75.0	1930	80.0
Yes	309	18.0	175	25.0	484	20.0
Total	1714	100.0	700	100.0	2414	100.0
High blood pressure						
No	1008	58.8	193	27.6	1201	49.8
Yes	706	41.2	507	72.4	1213	50.2
Total	1714	100.0	700	100.0	2414	100.0
High glucose						
No	1365	79.6	414	59.1	1779	73.7
Yes	349	20.4	286	40.9	635	26.3
Total	1714	100.0	700	100.0	2414	100.0
Metabolic syndrome						
No	1343	78.4	373	53.3	1716	71.1
Yes	371	21.6	327	46.7	698	28.9
Total	1714	100.0	700	100.0	2414	100.0
High uric acid						
No	1671	97.5	545	77.9	2216	91.8
Yes	43	2.5	155	22.1	198	8.2
Total	1714	100.0	700	100.0	2414	100.0
High ALT						
No	1475	86.1	341	48.7	1816	75.2
Yes	239	13.9	359	51.3	598	24.8
Total	1714	100.0	700	100.0	2414	100.0
High GGT						
No	1552	90.5	434	62.0	1986	82.3
Yes	162	9.5	266	38.0	428	17.7
Total	1714	100.0	700	100.0	2414	100.0
Glucose-lowering drugs						
No	1708	99.6	688	98.3	2396	99.3
Yes	6	0.4	12	1.7	18	0.7
Total	1714	100.0	700	100.0	2414	100.0
Triglyceride-lowering drugs						
No	1707	99.6	689	98.4	2396	99.3
Yes	7	0.4	11	1.6	18	0.7
Total	1714	100.0	700	100.0	2414	100.0
Cholesterol-lowering drugs						
No	1660	96.8	661	94.4	2321	96.1
Yes	54	3.2	39	5.6	93	3.9
Total	1714	100.0	700	100.0	2414	100.0
Blood pressure-lowering drugs						
No	1486	86.7	510	72.9	1996	82.7
Yes	228	13.3	190	27.1	418	17.3
Total	1714	100.0	700	100.0	2414	100.0

Table 3 reports the BICs, pseudo-R^2^ and AUC-ROC associated with the each of the 31 logistic regression models. Additional file 1: Table S1 gives the 31 multivariable logistic regression models developed for the analysis. Among the four parameters of interest, VAT was independently associated to all outcomes considered. In addition, log_e_VAT had the best combination of BIC, pseudo-R^2^ and ROC-AUC for high triglycerides (2056, 0.13, 0.75), high ALT (2198, 0.20. 0.80) and high GGT (1874, 0.18, 0.79). SAT was independently associated to MS and only with high blood pressure and high ALT when we considered the single biomarkers of MS and NAFLD. However, SAT never had the best combination of BIC, pseudo-R^2^ and ROC-AUC for anyone of the outcomes considered when compared to other parameters. The combination of log_e_VAT and SAT was associated with the best combination of BIC, pseudo-R^2^ and ROC-AUC for MS (2131, 0.28, 0.84). WC^−1^ was associated with the best combination of BIC, pseudo-R^2^ and ROC-AUC only for low HDL (2306, 0.06, 0.67) and high blood pressure (2576, 0.24, 0.81). The combination of pseudo-R^2^ and ROC-AUC shows a similar association between log_e_VAT and WC^−1^ with high glucose and high uric acid. Only BIC shows a marginal better association of such outcomes with WC^−1^.

**Table 3:** Association between the four parameters of interest and metabolic syndrome, its components, uric acid and altered liver enzymes

		Model 1	Model 2	Model 3	Model 4
		WC	logVAT	SAT	logVAT + SAT
High triglycerides	BIC	2085	2056	2175	2063
	Pseudo-R^2^ (McFadden)	0.12	0.13	0.08	0.13
	AUC-ROC	0.74	0.75	0.70	0.75
Low HDL	BIC	2306	2322	2418	2330
	Pseudo-R^2^ (McFadden)	0.06	0.05	0.01	0.05
	AUC-ROC	0.67	0.66	0.58	0.66
High blood pressure	BIC	2576	2677	2728	2623
	Pseudo-R^2^ (McFadden)	0.24	0.21	0.19	0.23
	AUC-ROC	0.81	0.79	0.78	0.80
High glucose	BIC	2327	2330	2463	2334
	Pseudo-R^2^ (McFadden)	0.18	0.18	0.13	0.18
	AUC-ROC	0.78	0.78	0.74	0.78
Metabolic syndrome	BIC	-	2158	2491	2131
	Pseudo-R^2^ (McFadden)	-	0.27	0.15	0.28
	AUC-ROC	-	0.83	0.76	0.84
High uric acid	BIC	1113	1117	1154	1125
	Pseudo-R^2^ (McFadden)	0.21	0.21	0.18	0.21
	AUC-ROC	0.84	0.84	0.84	0.81
High ALT	BIC	2252	2198	2350	2202
	Pseudo-R^2^ (McFadden)	0.18	0.20	0.14	0.20
	AUC-ROC	0.78	0.80	0.80	0.75
High GGT	BIC	1911	1874	2011	1888
	Pseudo-R^2^ (McFadden)	0.17	0.18	0.12	0.18
	AUC-ROC	0.78	0.79	0.74	0.79

Values are Bayesian information criterion (BIC), McFadden pseudo-R^2^ and areas under the ROC curve (AUC-ROC)

## Discussion

In the present study, we evaluated whether dissecting abdominal fat in VAT and SAT using US may detect stronger and more specific association with MS, MS components, hyperuricemia and altered liver enzymes compared to waist circumference. VAT was independently associated with all the outcomes of interest, while SAT was independently associated with MS and only with high blood pressure and high ALT when we considered the single parameters of MS and NAFLD. VAT had the strongest association with high triglycerides, high ALT and high GGT. The VAT + SAT association had the strongest association with MS. WC had the strongest association only with low HDL and high blood pressure. Lastly, VAT and WC were similarly associated to high glucose and high uric acid.

### High triglycerides

In keeping with the available evidence [2, 6, 22, 24–27], in the present study, an increasing VAT was associated with an increasing odds of hypertriglyceridemia. VAT was more strongly associated with high triglycerides than were SAT or WC, again in agreement with CT-based [2] and US-based [26] studies. While some studies found no association between SAT and hypertriglyceridemia [25, 26], other studies reported a weak association [2, 6, 27]. The association that we detected between SAT and hypertriglyceridemia disappeared after correction for VAT.

### Low HDL

In the present study, VAT and SAT were both associated with low HDL. However, SAT was associated with low HDL less strongly than VAT is in agreement with the previous studies [2, 6, 25–27]. Moreover, after adjustment for VAT, SAT lost its association with low HDL, suggesting that only VAT increment is involved in the decrement of serum HDL. Interestingly, contrary to previous studies [2, 26], we found that WC was more strongly associated with low HDL than was VAT. This finding may be partly due to the fact that we studied subjects at greater risk of CMD.

### High blood pressure

A previous association between VAT and hypertension has been reported by most [2, 6, 20, 25, 26] but not all [4, 27] studies. The existence of an association between SAT and hypertension is more controversial. We found a greater association with high blood pressure for VAT than for SAT. Interestingly, in agreement with previous studies [2, 6, 27], VAT and SAT were independently associated with high blood pressure. In addition, WC was associated with high blood pressure and more strongly than was VAT.

### High glucose

Consistently with previous studies [2, 6, 25, 26], the association of VAT with high glucose was higher than that of SAT. As reported by most [6, 25, 26] but not all [2] studies, there was no residual association between SAT and high glucose after the effect of VAT was controlled for. Interestingly, in the present study, VAT and WC were similarly associated to high glucose. It should be noted, indeed, that 1 of the 3 statistical association parameters considered showed a marginal better association of WC with high glucose whereas the others were superimposable to VAT.

### Metabolic syndrome

In the present study, both VAT and SAT were independently associated with MS. The VAT-MS association is in line with existing evidences. The contribution of SAT to MS is, instead, still controversial. Indeed, some [2], but not all [26], studies report a SAT-MS association. The contribution of SAT to MS in our subjects is likely to be due to the independent contribution of SAT to high blood pressure, which was the second most prevalent MS component in our subjects (50 %). Interestingly, the association of VAT and SAT with MS was greater when they were employed together than when they were used alone. Other researchers reported that US-determined VAT was strongly associated with MS in patients at risk of CMD [26]. However, they found no association of MS with SAT and did not evaluate the joint contribution of VAT and SAT to MS as we did in the present study [26].

### High ALT and high GGT

In a recent study performed in a large sample of subjects with and without NAFLD, VAT but not SAT was independently associated with high ALT [39]. However, the study did not compare WC and VAT in terms of their association with high ALT. In another study performed in overweight Korean women, CT-measured VAT was the only predictor of serum ALT at multivariable analysis controlling for SAT and other confounders [40]. A further study has confirmed that, after correction for confounders, ALT and GGT are higher in patients with increased VAT [41]. However, the association of WC with altered liver enzymes was not evaluated. In agreement with such studies, we found that VAT was associated with both high ALT and high GGT. In addition, we found that SAT was independently associated only with high ALT. Interestingly; the present study adds the information that VAT was associated more strongly than WC to high ALT and high GGT.

### High uric acid

In the present study, VAT and SAT were both associated with high uric acid. However, the association of SAT with high uric acid is lost when the effect of VAT is controlled for. An association between CT-measured VAT and uric acid was recently reported [9]. Such association persisted after SAT and other confounders were taken into account. A similar association between CT-measured VAT and uric acid has been reported among Japanese subjects [42, 43]. In agreement with these studies, the present study shows that VAT is associated more strongly to hyperuricemia than is SAT. Interestingly, VAT and WC were similarly associated to high uric acid. It should be noted, indeed, that only one of three statistical parameters considered in the present study reported a marginal better association of WC with high uric acid.

### Study strength and limitations

First strength of our study is its sample size. Second, although the comparison between WC and CT-measured abdominal fat in relation to the association with metabolic risk factors has been already studied [2], to our knowledge this is one of the first studies to compare WC with US measurements of abdominal fat distribution in relation to their associations with MS and its components, high uric acid, recently associated to MS [44–46], and altered hepatic enzymes as biomarkers of NAFLD. This study has, however, several limitations. First, we studied a self-selected sample of Caucasian subjects and our findings cannot be extrapolated to the general population and to non-Caucasian subjects. On the other hand, this is the largest study performed so far that has measured VAT and SAT by US and we believe that our findings are relevant for researchers interested into disentangling the effect of US-determined VAT and SAT on CMD risk. Second, this is a cross-sectional study. There is a general need of a cohort study aimed at evaluating the association of VAT and SAT changes with CMD risk changes. A large cross-sectional study with carefully standardized measurements of VAT and SAT, such as the present one, may help to plan such a study. Third, our outcomes (glucose, triglycerides, cholesterol, HDL-cholesterol, LDL-cholesterol, alanine transaminase, gamma-glutamyl-transferase and uric acid) were chosen because we considered them the most relevant biomarkers associated to metabolic syndrome and NAFLD. Never the less, we recognize that it could be interesting to study the association with some other important risk factors, such as total and LDL cholesterol, insulin, hemoglobin glycated and inflammatory parameters in particular stratifying for obesity degree. Finally, we used US to quantify abdominal VAT and SAT. Even though US is not a reference method, it is presently the only available option to measure abdominal fat in population studies. On the other hand, our US measurement protocol had been thoroughly validated against CT [14, 15] showing good accuracy and reproducibility and used in previous epidemiological studies [6, 47, 48]. In addition, all measurements were performed by the same operator, reporting a low coefficient of variation.

## Conclusion

In conclusion, US-determined VAT and SAT are both independently associated with MS. VAT is associated to all of the MS components in addition to hyperuricemia and altered liver enzymes, and performs equally or better than WC except for high blood pressure and low HDL. In contrast, SAT is independently associated only with high blood pressure and high ALT. These data are of clinical interest and suggest that the components of abdominal fat, as measured by US, may play at least in part independent roles in the development of cardiometabolic risk factors. Cohort studies are needed to test whether changes in US-measured VAT and SAT are associated with changes in the CMD risk profile.

## Additional file

Additional file 1: Table S1. Multivariable logistic regression models developed for studying the association between the 4 parameters of interest and the 8 outcomes considered. Describe how to calculate the probability of the outcome from each model. (PDF 370 kb)

## Abbreviation

ALT: Alanine transaminase
AUC-ROC: Area under the ROC curve
BIC: Bayesian information criterion
CMD: Cardiometabolic disease
CT: Computed tomography
CV: Coefficient of variation
GGT: Gamma-glutamyl-transferase
GOF: Goodness of fit
HL: Hosmer-Lemeshow test
MRI: Magnetic resonance imaging
MS: Metabolic syndrome
NAFLD: Non-alcoholic fatty liver disease
SAT: Subcutaneous adipose tissue
T2DM: Type 2 diabetes mellitus
US: Ultrasonography
VAT: Visceral adipose tissue
WC: Waist circumference
