# Supplementary material for: Metabolic correlates of subcutaneous and visceral abdominal fat measured by ultrasonography: a comparison with waist circumference
Source: Nutr J. 2016 Jan 6;15:2. doi: 10.1186/s12937-015-0120-2 (PMC4702394; doi:10.1186/s12937-015-0120-2)
Supplement: Additional file 1: Table S1. — Multivariable logistic regression models developed for studying the association between the 4 parameters of interest and the 8 outcomes considered. Describe how to calculate the probability of the outcome from each model. (PDF 370 kb) [file 12937_2015_120_MOESM1_ESM.pdf]

Additional file 1: Table S1

Multivariable logistic regression models developed for studying the association between the 4 parameters of interest and the 8 outcomes

	High triglycerides			
	Model 1<br>WC	Model 2<br>logVAT	Model 3<br>SAT	Model 4<br>logVAT + SAT
WC^{-1} (cm)	-448.02***<br>[-537.04 to -359.00]	—	—	—
Male gender	0.67***<br>[0.44 to 0.91]	0.61***<br>[0.36 to 0.85]	1.25***<br>[1.04 to 1.47]	0.62***<br>[0.37 to 0.88]
Age/10 (years)	0.15**<br>[0.05 to 0.25]	0.09<br>[-0.02 to 0.19]	0.30***<br>[0.21 to 0.39]	0.10<br>[-0.01 to 0.20]
$\log_e$VAT (cm)	—	1.62***<br>[1.31 to 1.93]	—	1.59***<br>[1.27 to 1.92]
SAT (cm)	—	—	0.17***<br>[0.09 to 0.26]	0.04<br>[-0.06 to 0.13]
Intercept	2.24***<br>[1.13 to 3.34]	-4.84***<br>[-5.40 to -4.28]	-3.72***<br>[-4.22 to -3.22]	-4.96***<br>[-5.55 to -4.36]
N	2414	2414	2414	2414

* $p < 0.05$, ** $p < 0.01$, *** $p < 0.001$ (Wald test)
Values are logistic regression coefficients (logits) and 95% confidence intervals

	Low HDL			
	Model 1<br>WC	Model 2<br>Logvat	Model 3<br>SAT	Model 4<br>logVAT + SAT
WC^{-1} (cm)	-446.75***<br>[-528.57 to -364.94]	—	—	—
Male gender	-0.18<br>[-0.42 to 0.06]	-0.16<br>[-0.40 to 0.09]	0.43***<br>[0.21 to 0.64]	-0.14<br>[-0.39 to 0.11]
Age/10 (years)	-0.19***<br>[-0.28 to -0.10]	-0.23***<br>[-0.32 to -0.13]	-0.03<br>[-0.11 to 0.06]	-0.22***<br>[-0.31 to -0.12]
Log_eVAT	—	1.35***<br>[1.08 to 1.62]	—	1.32***<br>[1.04 to 1.61]
SAT	—	—	0.17***<br>[0.09 to 0.25]	0.03<br>[-0.06 to 0.12]
Intercept	4.07***<br>[3.05 to 5.09]	-2.67***<br>[-3.14 to -2.20]	-1.90***<br>[-2.35 to -1.46]	-2.77***<br>[-3.28 to -2.25]
N	2414	2414	2414	2414

* $p < 0.05$, ** $p < 0.01$, *** $p < 0.001$ (Wald test)
Values are logistic regression coefficients (logits) and 95% confidence intervals.

	High blood pressure			
	Model 1<br>WC	Model 2<br>logVAT	Model 3<br>SAT	Model 4<br>logVAT + SAT
WC^{-1} (cm)	-606.56***<br>[-684.87 to -528.26]	—	—	—
Male gender	0.67***<br>[0.44 to 0.91]	0.83***<br>[0.59 to 1.06]	1.53***<br>[1.31 to 1.74]	0.99***<br>[0.75 to 1.23]
Age (years/10)	0.57***<br>[0.48 to 0.66]	0.54***<br>[0.45 to 0.63]	0.78***<br>[0.69 to 0.86]	0.63***<br>[0.54 to 0.72]
Log_eVAT (mm)	—	1.52***<br>[1.29 to 1.74]	—	1.25***<br>[1.01 to 1.48]
SAT (mm)	—	—	0.47***<br>[0.39 to 0.55]	0.34***<br>[0.25 to 0.42]
Intercept	3.96***<br>[3.00 to 4.92]	-4.80***<br>[-5.26 to -4.34]	-4.95***<br>[-5.44 to -4.45]	-5.80***<br>[-6.34 to -5.27]
N	2414	2414	2414	2414

* $p < 0.05$, ** $p < 0.01$, *** $p < 0.001$ (Wald test)
Values are logistic regression coefficients (logits) and 95% confidence intervals.

	High glucose			
	Model 1<br>WC	Model 2<br>logVAT	Model 3<br>SAT	Model 4<br>logVAT + SAT
WC^{-1} (cm)	-512.16***<br>[-598.06 to -426.26]	—	—	—
Male gender	0.38**<br>[0.15 to 0.61]	0.40***<br>[0.16 to 0.64]	1.06***<br>[0.86 to 1.26]	0.44***<br>[0.20 to 0.68]
Age (years/10)	0.52***<br>[0.43 to 0.61]	0.48***<br>[0.38 to 0.57]	0.67***<br>[0.58 to 0.76]	0.50***<br>[0.40 to 0.60]
Log_eVAT (mm)	—	1.58***<br>[1.30 to 1.85]	—	1.51***<br>[1.23 to 1.80]
SAT (mm)	—	—	0.22***<br>[0.14 to 0.30]	0.08<br>[-0.00 to 0.17]
Intercept	1.92***<br>[0.90 to 2.93]	-5.85***<br>[-6.43 to -5.27]	-4.89***<br>[-5.42 to -4.37]	-6.11***<br>[-6.75 to -5.47]
N	2414	2414	2414	2414

* $p < 0.05$, ** $p < 0.01$, *** $p < 0.001$ (Wald test)
Values are logistic regression coefficients (logits) and 95% confidence intervals

	Metabolic syndrome		
	Model 2<br>logVAT	Model 3<br>SAT	Model 4<br>logVAT + SAT
WC^{-1} (cm)	—	—	—
Male gender	0.17<br>[-0.07 to 0.41]	1.29***<br>[1.09 to 1.50]	0.32*<br>[0.07 to 0.56]
Age (years/10)	0.30***<br>[0.20 to 0.40]	0.65***<br>[0.56 to 0.74]	0.39***<br>[0.29 to 0.49]
Log_eVAT (mm)	2.87***<br>[2.54 to 3.20]	—	2.70***<br>[2.36 to 3.04]
SAT (mm)	—	0.46***<br>[0.37 to 0.54]	0.28***<br>[0.19 to 0.38]
Intercept	-7.14***<br>[-7.77 to -6.51]	-5.47***<br>[-5.98 to -4.95]	-8.15***<br>[-8.83 to -7.47]
N	2414	2414	2414

* $p < 0.05$, ** $p < 0.01$, *** $p < 0.001$ (Wald test)
Values are logistic regression coefficients (logits) and 95% confidence intervals

High uric acid				
	Model 1<br>WC	Model 2<br>logVAT	Model 3<br>SAT	Model 4<br>logVAT + SAT
WC^{-1} (cm)	-456.80***<br>[-606.76 to -306.83]	—	—	—
Male gender	1.85***<br>[1.45 to 2.24]	1.89***<br>[1.46 to 2.31]	2.40***<br>[2.05 to 2.75]	1.88***<br>[1.44 to 2.31]
Age (years/10)	0.17*<br>[0.03 to 0.31]	0.14<br>[-0.01 to 0.28]	0.30***<br>[0.16 to 0.43]	0.13<br>[-0.01 to 0.28]
Log_eVAT (mm)	—	1.32***<br>[0.83 to 1.82]	—	1.33***<br>[0.82 to 1.85]
SAT (mm)	—	—	0.07<br>[-0.05 to 0.19]	-0.02<br>[-0.15 to 0.11]
Intercept	0.47<br>[-1.27 to 2.22]	-6.35***<br>[-7.29 to -5.41]	-5.12***<br>[-5.88 to -4.35]	-6.29***<br>[-7.27 to -5.31]
N	2414	2414	2414	2414

* $p < 0.05$, ** $p < 0.01$, *** $p < 0.001$ (Wald test)
Values are logistic regression coefficients (logits) and 95% confidence intervals

	High ALT			
	Model 1<br>WC	Model 2<br>logVAT	Model 3<br>SAT	Model 4<br>logVAT + SAT
WC^{-1} (cm)	-467.96***<br>[-558.25 to -377.68]	—	—	—
Male gender	1.32***<br>[1.10 to 1.54]	1.24***<br>[1.02 to 1.46]	1.91***<br>[1.70 to 2.11]	1.29***<br>[1.06 to 1.51]
Age (years/10)	-0.06<br>[-0.15 to 0.03]	-0.14**<br>[-0.24 to -0.05]	0.12**<br>[0.04 to 0.21]	-0.11*<br>[-0.21 to -0.02]
Log_eVAT	—	1.80***<br>[1.51 to 2.10]	—	1.73***<br>[1.43 to 2.03]
SAT	—	—	0.24***<br>[0.16 to 0.33]	0.10*<br>[0.01 to 0.19]
Intercept	3.44***<br>[2.34 to 4.54]	-4.06***<br>[-4.56 to -3.57]	-3.06***<br>[-3.54 to -2.59]	-4.36***<br>[-4.93 to -3.79]
N	2414	2414	2414	2414

* $p < 0.05$, ** $p < 0.01$, *** $p < 0.001$ (Wald test)
Values are logistic regression coefficients (logits) and 95% confidence intervals

	High GGT			
	Model 1<br>WC	Model 2<br>logVAT	Model 3<br>SAT	Model 4<br>logVAT + SAT
WC^{-1} (cm)	-516.36***<br>[-616.94 to -415.79]	—	—	—
Male gender	1.17***<br>[0.93 to 1.41]	1.09***<br>[0.85 to 1.34]	1.79***<br>[1.57 to 2.02]	1.13***<br>[0.87 to 1.38]
Age (years/10)	-0.12*<br>[-0.22 to -0.02]	-0.21***<br>[-0.31 to -0.10]	0.07<br>[-0.02 to 0.16]	-0.18**<br>[-0.29 to -0.07]
logVAT	—	1.90***<br>[1.57 to 2.24]	—	1.85***<br>[1.50 to 2.19]
SAT	—	—	0.22***<br>[0.13 to 0.31]	0.07<br>[-0.02 to 0.17]
Intercept	3.73***<br>[2.52 to 4.93]	-4.45***<br>[-5.01 to -3.88]	-3.21***<br>[-3.72 to -2.71]	-4.68***<br>[-5.31 to -4.05]
N	2414	2414	2414	2414

* $p < 0.05$, ** $p < 0.01$, *** $p < 0.001$ (Wald test)
Values are logistic regression coefficients (logits) and 95% confidence intervals
